# Challenges and Solutions for Psychiatry’s Approach to Indigenous People

**DOI:** 10.1192/j.eurpsy.2025.388

**Published:** 2025-08-26

**Authors:** L. Mehl-Madrona, B. Mainguy

**Affiliations:** 1 Native Studies, University of Maine, Orono; 2 Psychiatry Residency, Northern Light Acadia; 3 Wabanaki Health and Wellness, Bangor, United States

## Abstract

**Introduction:**

Psychiatry has historically underserved Indigenous people. Earlier, cross-cultural psychiatry assumed that psychiatric disorders were universal and varied little across cultures. This approach has not worked well for Indigenous people who may have different views of mind and mental health. For example, Indigenous philosophy tend to explain the world and states of mental health from a storied approach encompassing relations to land, spiritual beings, ancestors, and the community which can result in different conclusions from conventional psychiatry.

**Objectives:**

We wanted to explore what modifications in their approach practicing psychiatrists have made to be successful in Indigenous communities and to determine what was common among how communities in which they worked conceptualized mind and mental health.

**Methods:**

We interviewed psychiatrists working in Indigenous communities regarding what was effective and how they had changed their practice to work in those communities and how those communities had changed them. We used the iterative processing of constructivist grounded theory to find commonalities in their responses. We present from rural and remote Indigenous settings in Canada (Saskatchewan and Northern Ontario), New Zealand, and Maine (USA).

**Results:**

We found a modified approach to psychiatric services that emphasized Indigenous values and that determined positive aspects of the client’s history as well as problem areas and engaged the client in therapy from the beginning of the evaluation. Some key concepts that emerged from qualitative analysis of interviews and case histories using constructivist grounded theory as a method of analysis included (1) reframing the person’s self-story within a threat-power-meaning network, (2) working with stories about the spirit of the suffering, (3) exploring right relationships and meaningful conduct, (4)acknowledging the intergenerational transmission of suffering. Physicians came to understand that the client sets their goals and defines what recovery means for them in discussion with their family and important community members including elders. This led to a different understanding of what privacy meant to clients. Indigenous cultures encountered were different but appeared to share some similarities including a highly relational approach to defining the self, a collectivist mindset in which the needs of the group can supersede the needs of the individual, a reliance upon stories for transmission of knowledge and culture, and a commitment to a biopsychosocial and spiritual approach.

**Conclusions:**

Psychiatry can form effective collaborative relationships with Indigenous communities requring modifications in the usual worldview and orientation to how psychiatry is practiced.

**Disclosure of Interest:**

None Declared

